# EXO1 inhibition selectively kills replication repair-deficient cancer cells

**DOI:** 10.1093/narcan/zcag029

**Published:** 2026-10-21

**Authors:** Marija Maric, Sarah E McClelland, Simon J Boulton

**Affiliations:** DSB Repair Metabolism Lab, The Francis Crick Institute, London NW1 1AT, United Kingdom; The Chromosomal Instability Lab, Centre for Cancer Evolution, Barts Cancer Institute, Queen Mary University of London, London EC1M 6BQ, United Kingdom; The Chromosomal Instability Lab, Centre for Cancer Evolution, Barts Cancer Institute, Queen Mary University of London, London EC1M 6BQ, United Kingdom; DSB Repair Metabolism Lab, The Francis Crick Institute, London NW1 1AT, United Kingdom

## Abstract

Deficiencies in DNA repair pathways create context-specific vulnerabilities that may be exploited therapeutically. Recent studies have established EXO1 as a druggable nuclease, but the genetic contexts that confer sensitivity to EXO1 inhibition remain incompletely defined. Here, we assessed whether pharmacological inhibition of EXO1 selectively targets repair-deficient cancer cells across multiple DNA repair backgrounds. Using potent and selective EXO1 inhibitors, we demonstrate impaired viability in cells deficient for Fanconi Anaemia pathway components (FANCG and APITD1/MHF1), BRCA1-A complex factor BRCC36, and the spliceosome factor ZRSR2. EXO1 inhibition in *FANCG* or *ZRSR2* KO cells induced accumulation of micronuclei, γH2AX, and RPA32 phosphorylation at serine 33, consistent with enhanced replication stress leading to cell death, and supporting a role for EXO1 in processing replication-associated DNA structures and maintaining genome stability under conditions of replication stress. Combining EXO1 inhibitor with PARP inhibitors exacerbated cytotoxicity, suggesting partially nonoverlapping repair functions and therapeutic potential for EXO1/PARP inhibitor combination strategies. Collectively, these findings establish that pharmacological inhibition of EXO1 recapitulates the sensitivity to genetic EXO1 loss in certain DNA repair deficient backgrounds, supporting its development as a targeted therapeutic strategy in molecularly stratified cancers.

## Graphical Abstract

**Graphical Abstract:**
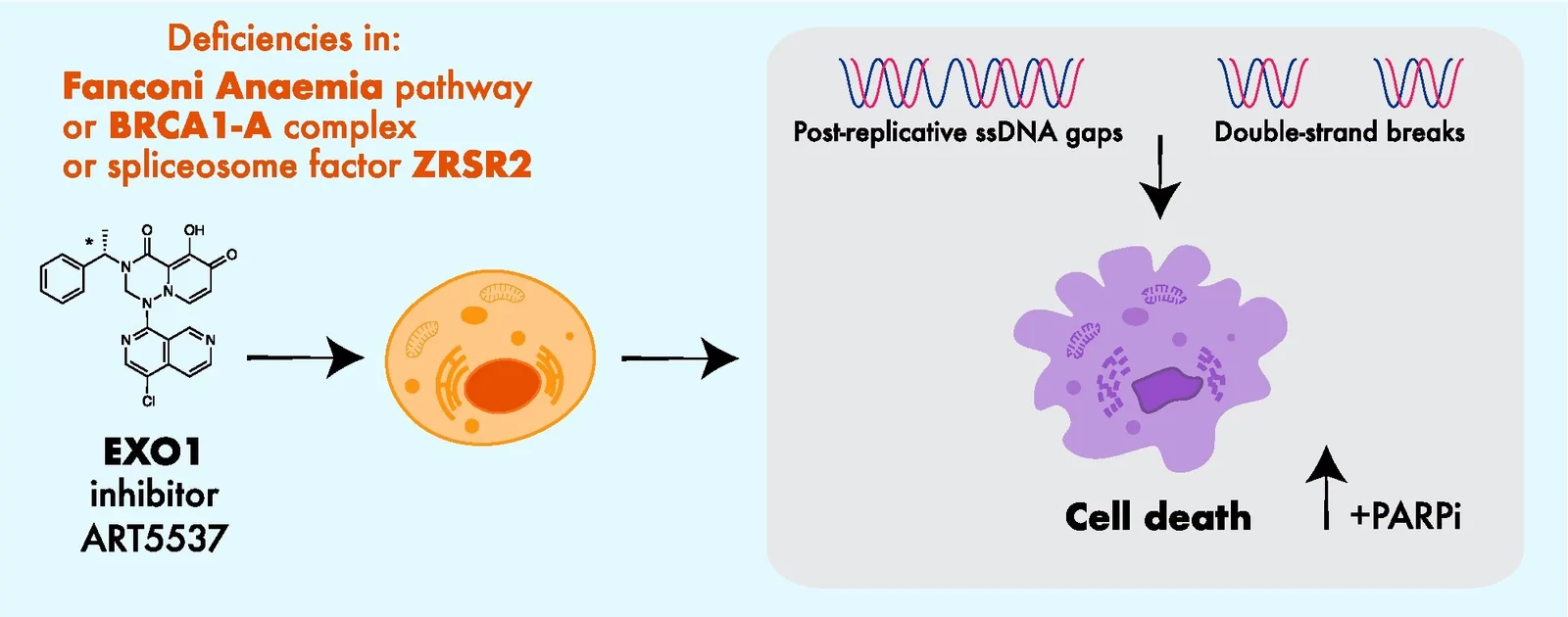

Impact statement Many cancers carry faults in the machinery that repairs DNA damage. Such faults can become a weakness: blocking a second piece of the repair machinery kills cancer cells while sparing healthy tissue. This study tested a new drug that blocks DNA repair component EXO1. Cells with faults in several repair pathways, including the Fanconi Anaemia pathway, were selectively killed. This effect increased when the drug was combined with PARP inhibitors, drugs already in clinical use. Tumour genetics could identify patients with DNA repair faults, who may benefit from EXO1 inhibitor treatment. The findings now require testing in animals and patients.

## Introduction

Genome instability is a defining hallmark of cancer and frequently arises through both germline and somatic alterations in DNA damage repair (DDR) pathways [1]. Large-scale cancer genomics studies have revealed that defects in DDR are not limited to canonical homologous recombination (HR) or mismatch repair pathways but extend across multiple DNA repair processes [2, 3]. However, despite this diversity, current therapeutic strategies target only a restricted subset of DDR deficiencies, most notably through PARP inhibition in HR-deficient tumours [4]. This creates a substantial gap between the prevalence of DDR alterations and the availability of actionable therapeutic strategies.

One approach to address this gap is the systematic discovery of context-specific genetic dependencies based on the identification of synthetic lethality genetic interactions [5]. In this context, genes classified as ‘strongly selective’ in DepMap datasets represent attractive therapeutic candidates, as they are nonessential in most contexts but critical for survival in defined genetic backgrounds. The structure-specific exonuclease EXO1 is one such factor that participates in multiple aspects of DNA metabolism, including mismatch repair [6], HR in double-strand DNA break (DSB) repair [7], Okazaki fragment processing [8], and replication fork processing [9]. Despite this breadth of function, EXO1 loss is generally well tolerated [10, 11], suggesting that its essentiality is conditional and dependent on compensatory pathways.

In our previous work, we systematically defined these compensatory dependencies through a genome-wide CRISPR dropout screen in EXO1-deficient cells [12]. This revealed a network of synthetic lethal interactions enriched for factors involved in replication-associated repair, most prominently the Fanconi Anaemia (FA) pathway, BRCA1-A complex components, and the spliceosome factor ZRSR2. Mechanistically, we demonstrated that these interactions converge on replication-coupled repair defects, characterized by impaired fork reversal, increased fork speeds, accumulation of postreplicative single-stranded DNA (ssDNA) gaps, and ultimately DSBs. Importantly, these phenotypes were causally linked to loss of viability, as modulation of replication dynamics through thymidylate synthase (TYMS) inhibition, reflected through decreased replication fork speed, could suppress lethality. While a recent study has identified small molecules targeting EXO1 catalytic activity [13], it remains unclear whether these compounds faithfully phenocopy genetic loss in relevant cancer contexts, particularly given the multifunctional roles of EXO1 that may not be fully captured by catalytic inhibition alone [14].

Here, using recently developed EXO1 inhibitors, we directly test whether pharmacological targeting of EXO1 is sufficient to exploit the genetic vulnerabilities identified in our prior work. We show that EXO1 inhibition selectively compromises viability in cells deficient for FA pathway components (FANCG, APITD1/MHF1), BRCA1-A complex (BRCC36), and spliceosome factor ZRSR2. We further demonstrate that these effects phenocopy genetic EXO1 loss at the level of replication stress, DNA damage accumulation, and cell death, and can be enhanced by PARP inhibition. Importantly, our data support a model in which EXO1 catalytic activity is specifically required to buffer replication-associated DNA processing in these deficient backgrounds, providing a mechanistic rationale for therapeutic targeting.

Collectively, this study functionally validates EXO1 as a druggable synthetic lethal target and establishes a framework for stratifying cancers based on replication-associated repair defects that confer sensitivity to pharmacological EXO1 inhibition.

## Materials and methods

### Cell lines

eHAP iCas9 cell lines [12], which have originally been generated from a diploidised eHAP parental cell line [15], were grown in IMDM media (Gibco) with 10% tetracycline-free fetal bovine serum (FBS; Pan-Biotech) and 1% penicillin/streptomycin (Gibco), at 37°C in 5% CO_2_. TOV-21G [16] and PL11 cell lines [17] were grown in RPMI 1640 media (Gibco) with 15% FBS (Sigma) and 1% penicillin/streptomycin (Gibco), at 37°C in 5% CO_2_.

### Compounds

Compounds developed for EXO1 inhibition [compound 20, compound 29 (ART5537), and compound 30] [13] were a generous gift from Artios Ltd, while ATM inhibitor (KU-55933), PARP inhibitor olaparib (AZD2281), PARP inhibitor veliparib (ABT-888), and TYMS inhibitor Pemetrexed (LY231514) were purchased from Selleckchem.

### siRNA transfections

ON-TARGETplus Nontargeting Control Pool siRNA and ON-TARGETplus SMARTPool Human BLM siRNA were purchased from Dharmacon and used for forward transfection with Lipofectamine RNAiMAX (Invitrogen) according to manufacturer’s instructions. Media was refreshed after 6 h and cells were collected for experiments 72 h after transfection.

### Inducible CRISPR knockouts

To induce Cas9 expression and CRISPR knockout in cell lines where guides have been integrated via lentiviral system, doxycycline was added to eHAP iCas9 cell lines at final concentration of 1 μg/ml for a total of 4 days before seeding for experiments.

### Clonogenic assay

Following the induction of CRISPR knockouts, cells were seeded into 24-well plates at the density of 200 cells/well. Compounds were added to cells immediately after seeding, resulting in four technical replicate wells per condition. After 6 days of growth, colonies were fixed and stained with 0.5% crystal violet solution with 20% methanol. Stained colonies were imaged with GelCount (Oxford Optronics), and GelCount software was used for well analysis. Colony numbers per well were averaged across technical replicates and averages were used to calculate ratios against the average of control wells for each cell line.

### CellTiter-Glo assay

eHAP iCas9 cells were seeded in opaque 96-well plates at the density of 200 cells/well either 72 h after transfection (for experiments with siRNA-mediated silencing) or 96 h after doxycycline-Cas9 induction (for experiments with inducible CRISPR KOs). TOV-21G and PL11 cells were seeded in opaque 96-well plates at the density of 4000 and 1000 cells/well, respectively. Compounds were added to the media the following day, and after 5 days of treatment, wells were processed with CellTiter-Glo 2.0 reagent (Promega) following manufacturer’s protocol. Luminescence signal for each well was acquired with CLARIOstar Plus Microplate Reader (BMG Labtech) or FLUOstar Omega Microplate Reader (BMG Labtech). Values for each well were normalized against untreated control well, or against the control well with fixed concentration of PARP or ATM inhibitor.

### Incucyte Cytotox cell death assay

To measure the rate of cell death following addition of ART5537, 5000 cells were seeded in an opaque 96-well plate. The following day Incucyte Cytotox green dye (Sartorius) was added to cells at a final concentration of 5 nM alongside appropriate treatments. After 48 h of treatment wells were scanned on EVOS M700 Imaging System (Invitrogen) using 4× objective to acquire green signal. Dead cells in each well were quantified with Celleste 6 Image Analysis Software (Invitrogen) and signal was normalized against untreated cells for each cell line for each ART5537 concentration.

### Analysis of protein depletion by immunoblotting

#### Cell extracts preparation

eHAP iCas9 cells were harvested following 4 days of doxycycline treatment, 72 h of siRNA silencing or following 24-h treatment with ART5537. Cells were counted and 2 × 10^5^ cells were pelleted at 2300 × *g* for 5 min, followed by a wash in phosphate buffered saline (PBS). Washed cells were again pelleted and pellets were frozen at −80°C until ready for lysate preparation. To make protein lysates, cells were resuspended in 50 μl of modified Laemmli buffer [50 mM Tris–HCl, pH 6.8, 100 mM dithiothreitol, 2% sodium dodecyl sulphate (SDS), 0.1% bromophenol blue, 10% glycerol]. Chromatin was digested with Benzonase (Millipore) by 30-min incubation at 25°C with shaking at 1000 rpm in Thermomixer (Eppendorf). Samples were then boiled for 10 min at 95°C, before immunoblotting analysis.

#### Immunoblotting

Protein lysates were resolved in NuPAGE sodium dodecyl sulphate–polyacrylamide gel electrophoresis (SDS–PAGE) gels (Invitrogen, 4%–12% Bis–Tris, 12% Bis–Tris, or 3%–8% Tris–acetate gels) and transferred to nitrocellulose membrane (0.45 μm, Thermo Scientific). Membranes were blocked in 5% skimmed milk (VWR) in TBST buffer, before incubating with primary antibodies and secondary antibodies (both in TBST buffer with 5% skimmed milk). Primary antibodies used: anti-BLM (ab2179, Abcam, rabbit polyclonal), anti-FANCG (F-8, sc-393382, Santa Cruz, mouse monoclonal), anti-BRCC36 (A302-517A-M, Bethyl Laboratories, rabbit polyclonal), anti-vinculin (66305-1-Ig, ProteinTech, mouse monoclonal), anti-alpha-tubulin (clone B-5-1-2, T6074, Sigma, mouse monoclonal), anti-KAP1-pS824 (ab70369, Abcam, rabbit polyclonal), anti-KAP1 (clone 20C1, ab22553, Abcam, mouse monoclonal), anti-CHK2-pT68 (2661S, Cell Signaling Technologies, rabbit polyclonal), anti-CHK2 (clone 7, 05-649, Millipore, mouse monoclonal), and anti-beta-actin HRP conjugate (clone 13E5, 5125, Cell Signaling Technologies, rabbit monoclonal). Anti-APITD1/MHF1 antibody (rabbit polyclonal) was a kind gift from Weidong Wang [18]. Secondary antibodies used were anti-mouse IgG, HRP-linked antibody (7076S, Cell Signaling Technology), and anti-rabbit immunoglobulins/HRP (Dako). Chemiluminescence signal was acquired on Bio-Rad ChemiDoc MP following incubation with Clarity Western ECL, Clarity Max Western ECL (both Bio-Rad), or Immobilon Forte Western HRP Substrate (Sigma Aldrich).

### Sequencing of inducible *ZRSR2* knockout cell line and CRISPR editing analysis

Analysis of the target locus editing was performed by isolation of genomic DNA from cells harvested after 4 days of Cas9 expression induction using QuickExtract DNA Extraction Solution (Biosearch Technologies), followed by polymerase chain reaction (PCR) with Q5 High-Fidelity DNA Polymerase (NEB) according to manufacturer’s instructions and using oligonucleotides for specific amplification of *ZRSR2* targeted locus (ZRSR2 KO FW check: CTGTCTGTGGTGGTTCCCTA, ZRSR2 KO REV check: CCACCCAGGGAACAAGGATA). PCR reactions were purified and sequenced by Source BioScience. Sequencing traces were analysed with ICE CRISPR Analysis Tool (EditCo) to determine editing efficiency at *ZRSR2* sgRNA (AGTTGGAAAATGGTACCACA) cut site.

### Immunofluorescence staining and high-throughput imaging

#### Sample preparation for immunofluorescence experiments

Cells were seeded at 5000 cells/well in 96-well PhenoPlate (Revity), and ART5537 in respective final concentrations was added to the media the following day. After 24 h of treatment, cells were washed with PBS, fixed with PTEMF buffer (0.2% Triton X-100, 20 mM PIPES, pH 6.8, 10 mM EGTA, 1 mM MgCl_2_, and 4% formaldehyde) for 10 min at room temperature, and rinsed with PBS three times before storage at 4°C.

#### Immunofluorescence

All biological replicates were stained in parallel. Fixed cells were incubated in 3% bovine serum albumin PBS solution for all steps during staining. Anti-phospho-histone H2A.X (Ser139) antibody (clone JBW301, 05-636, Millipore, mouse monoclonal) primary antibody was used for γH2AX staining, while anti-RPA-pS33 (A300-246A, Bethyl Laboratories) primary antibody was used for phosphorylated (Ser33) RPA32 staining. Goat anti-rabbit Alexa Fluor 594 (A11037, Invitrogen) and goat anti-mouse Alexa Fluor 488 (A11029, Invitrogen) were used as secondary antibodies. Following immunostaining, cells were washed in PBS three times, and DNA was stained with DAPI at final concentration 1 μg/ml during the second PBS wash. Samples were kept in PBS at 4°C until imaging.

#### High-throughput imaging

Wells were imaged with laser confocal system IN Cell Analyzer 6000 (GE Healthcare) using 40× objective (NA 0.6). Acquired images were analysed with INCarta software (GE Healthcare).

### Quantifications and statistical analyses

Each replicate used for analyses was a biological replicate, with graphs showing means with standard deviation. Statistical analyses were performed using GraphPad Prism software (version 11), with analyses and corresponding tests noted in legends.

## Results

### A potent inhibitor of human exonuclease EXO1 catalytic activity mimics genetic loss of EXO1 in human cells

Our prior CRISPR-based genetic screens established that EXO1 loss creates critical dependencies on parallel DNA repair pathways, particularly those involved in long-range resection and replication fork processing [12]. However, a key unresolved question is whether pharmacological inhibition of EXO1 catalytic activity is sufficient to recapitulate these dependencies, or whether non-catalytic functions of EXO1 contribute to the observed synthetic lethal interactions.

To address this, we first focused on previously documented synthetic lethal interactions [12, 19, 20] within DSB repair, where role of EXO1 is well described. Inhibition of ATM kinase, the central kinase in DSB repair activation, has been reported as synthetic lethal with EXO1 loss in a genome-wide CRISPR screen in lung cancer cells with an ATM inhibitor [20]. ATM loss was also observed in our *EXO1* KO CRISPR dropout screen as a synthetic lethal genetic interaction and then validated pharmacologically in diploid eHAP iCas9 cells [12]. Similarly, EXO1 and the BLM–DNA2 pathway function redundantly in long-range DNA end resection [7, 19]. Therefore, these interactions provide a model for comparison of genetic and pharmacological perturbation of EXO1.

To investigate the utility of a recently developed catalytic inhibitor of EXO1 [13] (compound 20; EXO1 IC_50_ 13 nM; Fig. 1A), we examined the impact of ATM inhibition (KU-55933) in combination with EXO1 compound 20. Treatment of eHAP iCas9 cells with compound 20 and ATM inhibitor resulted in a dose-dependent reduction in viability relative to EXO1 inhibitor alone in wild-type background (Fig. 1B), consistent with previously reported genetic interaction data. To investigate the effect of EXO1 inhibition on BLM-deficient cells, we depleted BLM helicase using siRNA (Supplementary Fig. S1A and B) and treated either BLM-depleted eHAP iCas9 cells or cells transfected with non-targeting siRNA with compound 20. BLM-depleted eHAP iCas9 cells exhibited increased sensitivity to compound 20, relative to control cells (Supplementary Fig. S1C), indicating a specific effect of EXO1 inhibition on the viability of cells lacking BLM.

**Figure 1.**
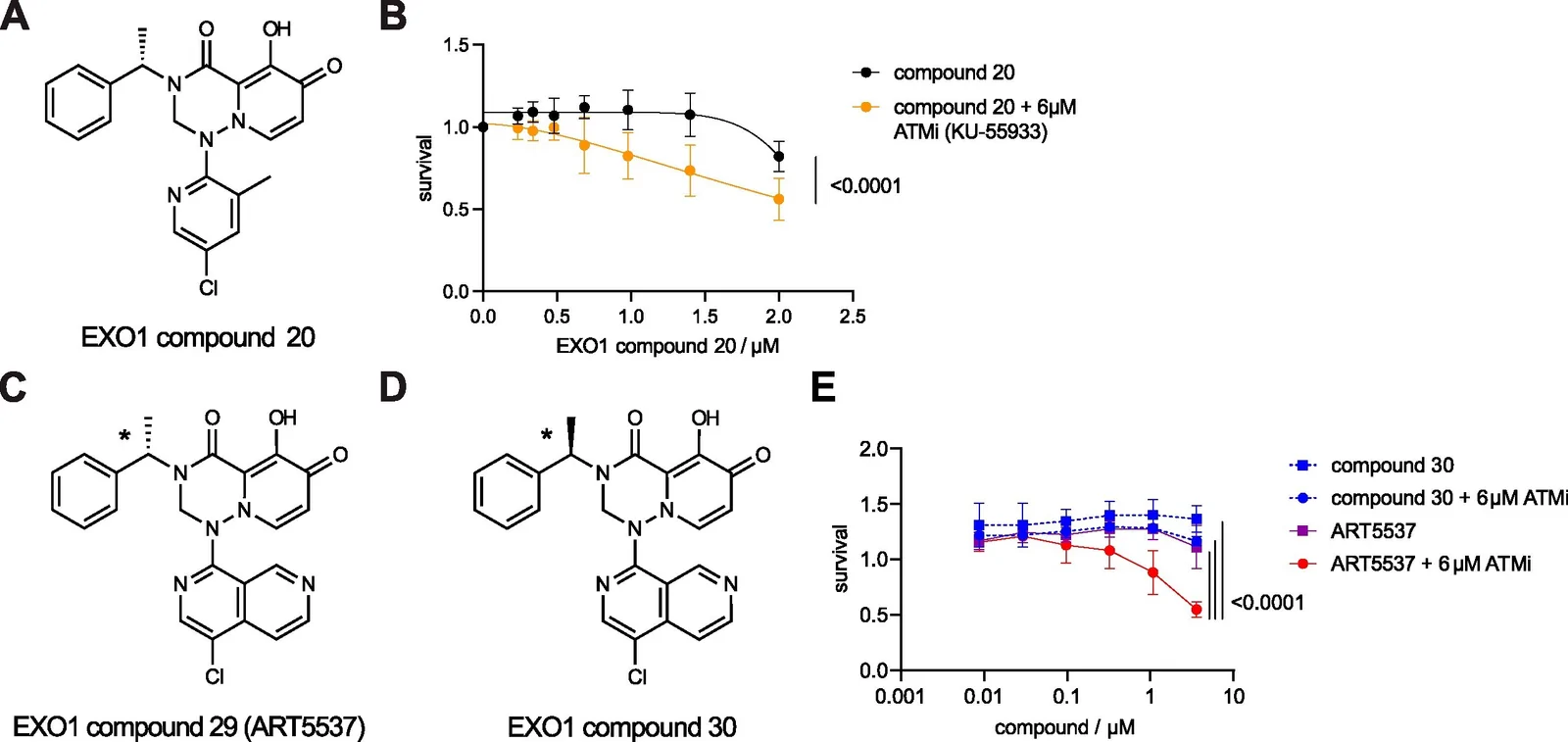
(**A**) Chemical structure of EXO1 compound 20 [13]. (**B**) Survival of eHAP iCas9 cells treated with EXO1 compound 20 without or with ATM inhibitor KU-55933 (*n* = 3); ordinary two-way ANOVA with Šidák’s test. (**C**) Chemical structure of EXO1 compound 29, ART5537 [13]. (**D**) Chemical structure of the less active enantiomer EXO1 compound 30 [13]. (**E**) Survival of eHAP iCas9 cells treated with ART5537 or compound 30 without or with ATM inhibitor KU-55933 (*n* = 3); ordinary two-way ANOVA with Tukey’s test.

To confirm our findings, we utilized the most potent available EXO1-inhibiting compound ART5537 (compound 29, EXO1 IC_50_ 3.4 nM, Fig. 1C), with the less active enantiomer compound 30 as a control molecule (EXO1 IC_50_ 344 nM, Fig. 1D) [13]. Similarly to the combination of ATM kinase inhibitor with compound 20, treatment with EXO1-inhibiting compound ART5537 together with ATM kinase inhibitor showed a significant decrease in survival of eHAP iCas9 cells in comparison to survival after treatment with ART5537 alone, or compound 30 with and without ATM kinase inhibition (Fig. 1E). Importantly, the magnitude of sensitization was comparable to that observed in genetic perturbation experiments [12], supporting the on-target activity of the compound. However, it is important to note that these combinatorial effects do not, by themselves, exclude broader exacerbation of replication stress as an alternative explanation.

Together, these data support the conclusion that EXO1 catalytic inhibition is functionally sufficient to recapitulate at least a subset of genetic synthetic lethal interactions, particularly those linked to DNA end processing and damage signalling.

### Pharmacological inhibition of EXO1 recapitulates synthetic lethality with EXO1 loss and inducible loss of FANCG, APITD1/MHF1, BRCC3/BRCC36, and ZRSR2, and sensitizes cancer cell lines with FA factor deficiencies

Building on the genetic framework established previously, we next examined whether pharmacological EXO1 inhibition can target DDR deficiencies frequently mutated in cancer identified in our *EXO1* KO CRISPR screen. These include components of the FANCM complex, FA core complex, BRCA1-A complex, and spliceosome factor ZRSR2 (Fig. 2A) [12]. Collectively, somatic mutations of these factors are observed in 8.49% of all cancers, representing a sizeable opportunity for targeted intervention (Supplementary Fig. S2A). Notably, homozygous deletions of *FANCG, APITD1, BRCC3*, and *ZRSR2* are found in 10.41% mature B-cell neoplasms and 5.49% adrenocortical carcinomas (Supplementary Fig. S2B). The FANCM complex and FA core complex function at the apex of the Fanconi Anaemia signalling pathway, which responds to replication stress and coordinates interstrand crosslink (ICL) repair [21–28]. BRCA1-A complex is a specific BRCA1-associated complex required to recruit BRCA1 to DSB sites [29–31]. The spliceosome factor ZRSR2 is a bona fide cancer driver [32, 33], whose loss exhibits hallmarks of Fanconi Anaemia deficiency, including compromised FA pathway activation, increased genome instability, and a hypomorphic response to ICL crosslinkers when compared to an FA gene knockout [12].

**Figure 2.**
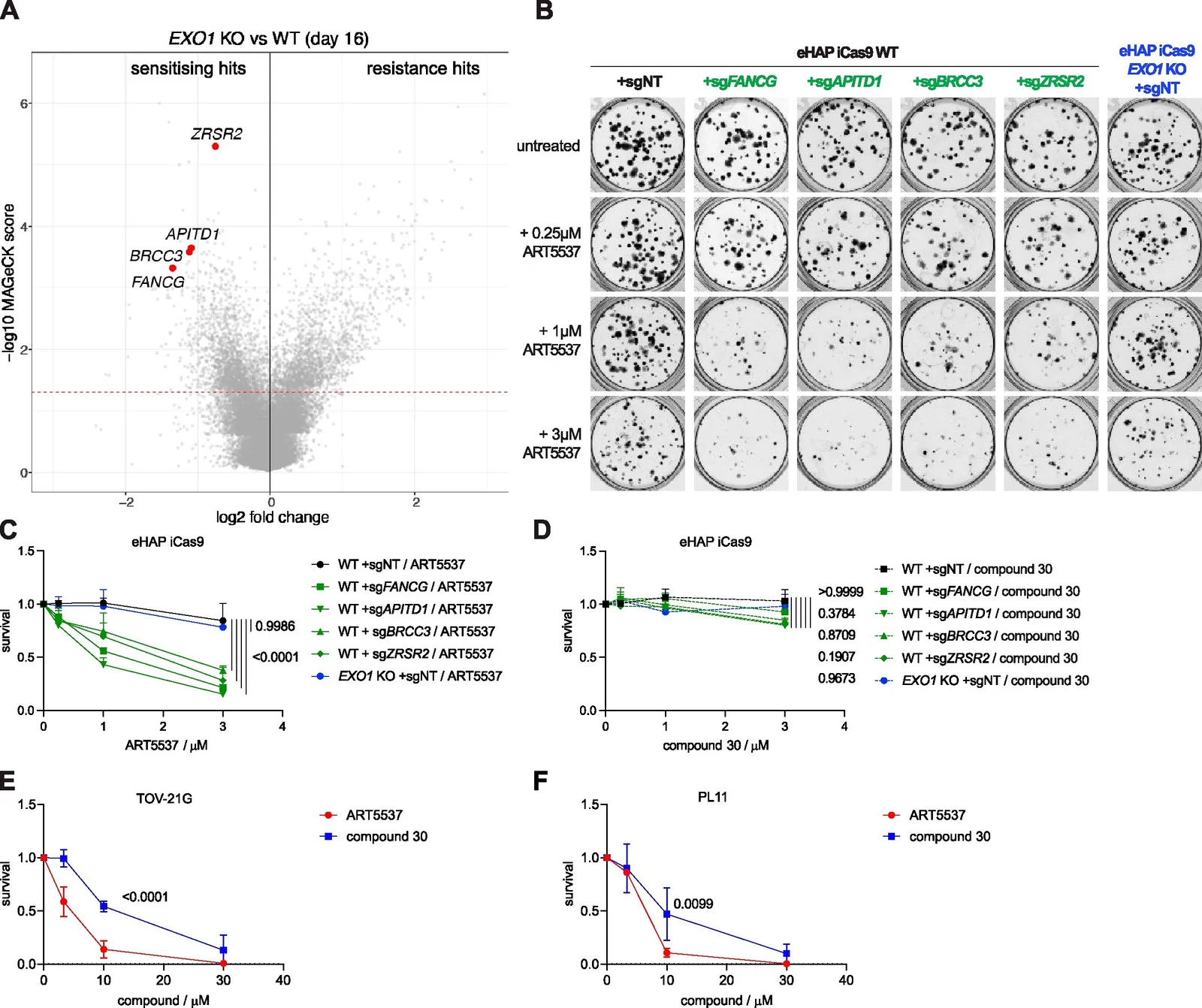
(**A**) Volcano plot with selected cancer-relevant synthetic lethal interactions in genome-wide CRISPR dropout screen in eHAP iCas9 *EXO1* KO cells (day 16) [12]. (**B**) Representative images of wells from clonogenic assay following ART5537 treatment upon induction of KO in eHAP iCas9 cells with integrated sgRNAs. (**C**) Quantification of clonogenic assay of eHAP iCas9 inducible KO cell lines with ART5537 (*n* = 3); ordinary two-way ANOVA with Tukey’s test. (**D**) Quantification of clonogenic assay of eHAP iCas9 inducible KO cell lines with compound 30 (*n* = 3); part of the experiment shown in panels (B) and (C), statistical analysis same as in panel (C). (**E**) Survival of patient-derived cancer cell line TOV-21G treated with ART5537 or compound 30 *(n* = 3); ordinary two-way ANOVA with Šidák’s test. (**F**) Survival of patient-derived cancer cell line PL11 treated with ART5537 or compound 30 (*n* = 3); ordinary two-way ANOVA with Šidák’s test.

To establish whether pharmacological inhibition of EXO1 sensitizes cells with deficiencies in FA core complex, FANCM complex, BRCA1-A complex, or ZRSR2, we utilized EXO1-inhibiting compound ART5537 and compound 30 as a control molecule (Fig. 1C and D). These compounds were tested in our previously established system in diploid eHAP iCas9 cells [12, 15], which possess an integrated inducible Cas9 construct and the gene-targeting sgRNA sequence. In these cell lines the respective sgRNA is constitutively expressed, which leads to knockout in ∼90% eHAP iCas9 cells upon Cas9 expression induction. Cell lines with integrated sgRNAs for *FANCG, APITD1, BRCC3*, and *ZRSR2* were subjected to doxycycline for 4 days to induce Cas9 expression and knockout of the respective genes (Supplementary Fig. S2C–G). Following induction of KOs, cells were treated for 5 days with either ART5537 or control compound 30 and surviving colonies were assessed by clonogenic assay.

Treatment with EXO1 inhibitor ART5537 resulted in a marked reduction in clonogenic survival in cells lacking FANCG, APITD1/MHF1, BRCC3/BRCC36, or ZRSR2, while wild-type and *EXO1* KO cells were largely unaffected (Fig. 2B and C, and Supplementary Fig. S3). In contrast, the control enantiomer compound 30 had minimal impact on cell survival, irrespective of genetic background (Fig. 2D and Supplementary Figs S2H and S3). Notably, the degree of sensitivity observed in ART5537-treated cells closely mirrors that of genetic *EXO1* knockout backgrounds, suggesting that catalytic inhibition is sufficient to phenocopy the synthetic lethal phenotype. These results show that EXO1 inhibition specifically induces loss of viability in cells lacking functional FA pathway, BRCA1-A complex, or spliceosome factor ZRSR2, in a manner that is comparable to the genetic loss of EXO1.

To address whether findings from the eHAP isogenic system are representative of patient-derived cancer cell lines harbouring FA deficiencies, we examined cell survival following treatment with either ART5537 or compound 30 in FANCF-deficient ovarian cancer cell line TOV-21G [16] and in FANCC-deficient pancreatic adenocarcinoma cell line PL11 [17]. In both cell lines treatment with ART5537 significantly reduced cell survival in comparison to treatment with the control compound 30 (Fig. 2E and F), demonstrating that catalytic inhibition of EXO1 can sensitize cancer cells with FA pathway deficiencies.

### Constitutive loss of FANCG and ZRSR2 leads to cell death upon EXO1 inhibition

To examine how pharmacological inhibition of EXO1 affects cells in which loss of FA pathway and ZRSR2 has been established, we utilized constitutive KOs of *FANCG* and *ZRSR2* in eHAP iCas9 cells that we have previously derived and validated [12]. Treatment of eHAP iCas9 *FANCG* and *ZRSR2* KOs with ART5537 conferred a significant loss of viability in comparison to either eHAP iCas9 wild-type cells or in comparison to treatment with the control compound 30 (Fig. 3A).

**Figure 3.**
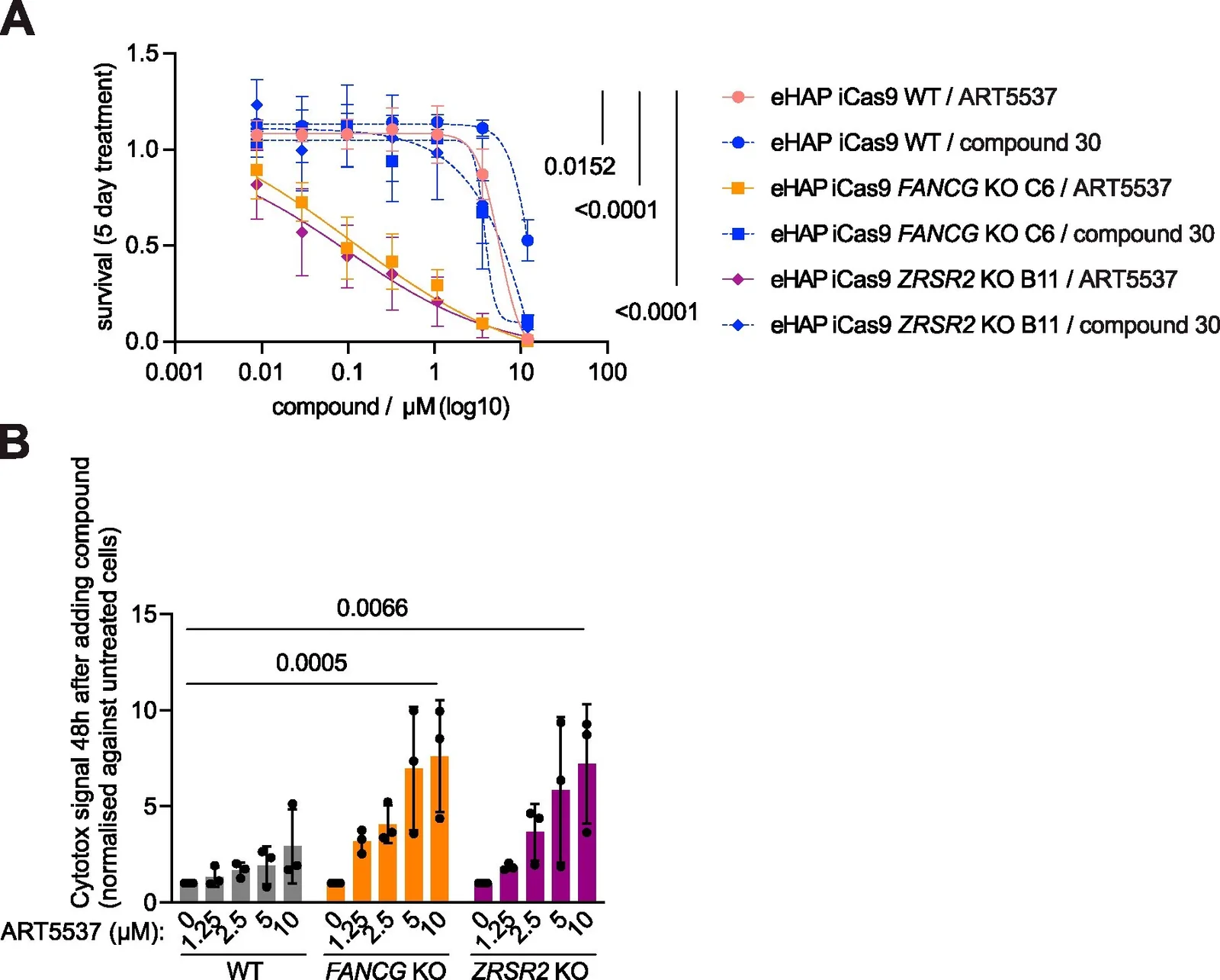
**(A**) Survival of eHAP iCas9 wild type, *FANCG* KO, and *ZRSR2* KO cells following a 5-day treatment with either ART5537 or compound 30; ordinary two-way ANOVA with Tukey’s test. For eHAP WT ART5537 versus compound 30 comparison, *P*-value is expressed below the short-sized bar, while *P*-values for comparisons between eHAP WT and *FANCG* KO or WT and *ZRSR2* KO treated with ART5537 are expressed below the medium-sized and long-sized bars, respectively. (**B**) Cytotox cell death assay for eHAP iCas9 wild type, *FANCG* KO, and *ZRSR2* KO cells following 48 h of ART5537 treatment; ordinary two-way ANOVA with Dunnett’s test.

As we have previously shown that genetic loss of EXO1 with cancer-relevant synthetic lethal hits is due to apoptotic cell death [12], we examined whether pharmacological inhibition of EXO1 also leads to cell death. To this end, eHAP iCas9 wild type, *FANCG* KO, and *ZRSR2* KO cells were seeded before the addition of Cytotox dye and ART5537 to the media. Cytotox-labelled dead cells were quantified 2 days after the addition of dye and ART5537, which revealed a dose-dependent increase of dead cells in comparison to untreated cells for both *FANCG* and *ZRSR2* KO cell lines (Fig. 3B). These data show that the EXO1 inhibitor ART5537 specifically induces cell death in cells lacking FANCG and ZRSR2.

### EXO1 inhibition in eHAP iCas9 *FANCG* and *ZRSR2* KO cells induces genome instability, increased DSB formation, and increase in replication stress response

Synthetic lethality between EXO1 and either FANCG or ZRSR2 has been reported to result in increased genomic instability associated with elevated replication stress [12]. To determine whether the observed cell death induced by ART5537 in eHAP iCas9 *FANCG* KO and *ZRSR2* KO cells is similarly driven by the induction of genomic instability, we quantified markers of DNA damage and replication stress in eHAP iCas9 wild-type, *FANCG* KO, and *ZRSR2* KO cells 24 h after treatment with ART5537 using high-throughput imaging.

First, we assessed whether ART5537 induces micronuclei, a marker associated with replication stress and chromosome mis-segregation. At concentrations of ART5537 (2.5 and 5 μM) that strongly reduced the viability of *FANCG* KO and *ZRSR2* KO cells, but not wild-type cells (Fig. 3A), both knockout cell lines exhibited a significant increase in the proportion of cells containing micronuclei after 24 h of drug treatment (Fig. 4A and B). In contrast, wild-type cells showed only a small, non-significant increase, comparable to the baseline levels observed in untreated *FANCG* KO and *ZRSR2* KO cells.

**Figure 4.**
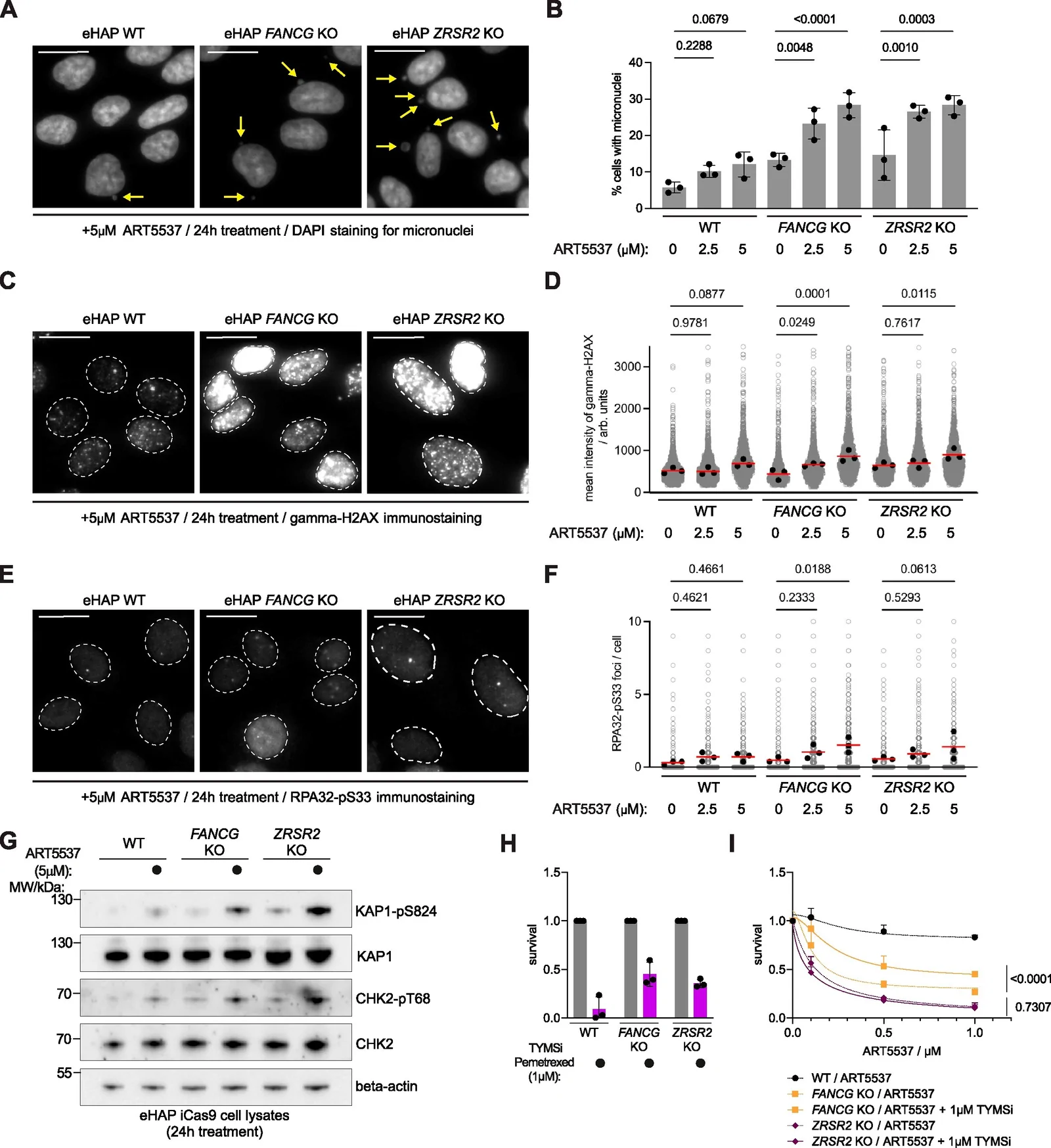
**(A**) Representative images for micronuclei induced by 24-h ART5537 treatment in eHAP iCas9 wild type, *FANCG* KO, and *ZRSR2* KO cells. Yellow arrows point to each micronucleus. Scale bar: 20 μm. (**B**) Quantification of cells with micronuclei for eHAP iCas9 wild type, *FANCG* KO, and *ZRSR2* KO cells in untreated condition or after 24-h treatment with either 2.5 or 5 μM ART5537 (*n* = 3, quantification based on ≥399 nuclei per condition). Ordinary two-way ANOVA was performed on means from each biological replicate, with *P*-values denoted above columns. Mean with standard deviation is shown for each sample based on mean of the biological replicates. (**C**) Representative images for γH2AX signal induced by 24-h ART5537 treatment in eHAP iCas9 wild type, *FANCG* KO, and *ZRSR2* KO cells. Nuclei borders are denoted with dotted lines. Scale bar: 20 μm. (**D**) Quantification of mean intensity of γH2AX in eHAP iCas9 wild type, *FANCG* KO, and *ZRSR2* KO cells in untreated condition or after 24-h treatment with either 2.5 or 5 μM ART5537 (*n* = 3, quantification based on ≥397 nuclei per condition). Ordinary two-way ANOVA was performed on means from each biological replicate (red line denotes overall mean), with *P*-values denoted above columns. (**E**) Representative images for RPA32-pS33 foci induced by 24-h ART5537 treatment in eHAP iCas9 wild type, *FANCG* KO, and *ZRSR2* KO cells. Nuclei borders are denoted with dotted lines. Scale bar: 20 μm. (**F**) Quantification of RPA-pS33 foci in eHAP iCas9 wild type, *FANCG* KO, and *ZRSR2* KO cells in untreated condition or after 24-h treatment with either 2.5 or 5 μM ART5537 (*n* = 3, quantification based on ≥397 nuclei per condition). Ordinary two-way ANOVA was performed on means from each biological replicate (red line denotes overall mean), with *P*-values denoted above columns. (**G**) Immunoblotting of cell extracts from eHAP iCas9 wild type, *FANCG* KO, and *ZRSR2* KO cells following 24-h treatment with 5 μM ART5537. Samples were probed for phosphorylated levels of KAP1 and CHK2 with controls, and for beta-actin as loading control. (**H**) Quantification of clonogenic assay of eHAP iCas9 wild type, *FANCG* KO, and *ZRSR2* KO cell lines with 1 μM TYMS inhibitor Pemetrexed (*n* = 3). (**I**) Survival of eHAP iCas9 wild type, *FANCG* KO, and *ZRSR2* KO cells treated with ART5537 without or with TYMS inhibitor Pemetrexed (*n* = 3); ordinary two-way ANOVA with Tukey’s test.

We next examined γH2AX (phosphorylated H2AX at serine 139), a marker of DSB. While 24-h treatment with 5 μM ART5537 resulted in only a modest, non-significant increase in γH2AX signal in wild-type cells, the mean γH2AX intensity was significantly increased in both *FANCG* KO and *ZRSR2* KO cells compared to their respective untreated controls (Fig. 4C and D). These results indicate that FANCG- and ZRSR2-deficient cells accumulate higher levels of DNA damage following EXO1 inhibition, consistent with previously reported synthetic lethal interactions between EXO1 and either FANCG or ZRSR2 [12].

As our previous mechanistic analyses of these synthetic lethal interactions also revealed increased replication stress, evidenced by elevated phosphorylation of RPA32 at serine 33 (RPA32-pS33) in both contexts, as well as increased ssDNA gaps at replication forks in *EXO1*-*FANCG* double KO cells, we next investigated whether pharmacological inhibition of EXO1 recapitulates this phenotype in cells lacking FANCG or ZRSR2. Quantification of RPA32-pS33 foci following 24-h ART5537 treatment showed increased numbers of foci in both *FANCG* KO and *ZRSR2* KO cells relative to untreated controls, with levels exceeding those observed in treated wild-type cells (Fig. 4E and F).

We further confirmed by immunoblotting that eHAP iCas9 *FANCG* KO and *ZRSR2* KO cells following 24-h ART5537 treatment exhibited elevated DSB signalling in comparison to eHAP iCas9 wild-type cells and untreated controls (Fig. 4G). ATM-dependent phosphorylation of KAP1 and CHK2 were significantly elevated in both KO cell lines following 24-h EXO1 inhibition, indicative of increased DNA damage driven by ART5537 treatment in the absence of FANCG or ZRSR2 and in agreement with our earlier genetic observations [12].

Finally, we investigated whether thymidylate synthase (TYMS) inhibition can suppress sensitivity of *FANCG* KO and *ZRSR2* KO cells to EXO1 inhibition, similarly to our previous observations upon EXO1 loss. *TYMS* was a hit in genome-wide CRISPR rescue screens in *EXO1-FANCG* and *EXO1-ZRSR2* double KO cell lines, which were then shown to be resistant to treatment with TYMS inhibitor Pemetrexed at concentrations that were sufficient to kill wild-type cells [12]. Comparably to the inducible eHAP iCas9 system [12], constitutive eHAP iCas9 KOs of both *FANCG* and *ZRSR2* exhibited only a moderate loss of viability with 1 μM Pemetrexed, which is sufficient for near-complete loss of viability of wild type eHAP iCas9 cells (Fig. 4H). While *ZRSR2* KO cells responded similarly to EXO1 inhibition with and without Pemetrexed, Pemetrexed-treated *FANCG* KO cells exhibited improved survival to EXO1 inhibition in comparison to survival rate with EXO1 inhibition alone (Fig. 4I). These findings suggest that lethality of *FANCG* KO cells following EXO1 inhibition can at least partially be attributed to modulated replication dynamics, further confirming similarities with genetic findings with *EXO1-FANCG* double KO.

Taken together, these results demonstrate that pharmacological inhibition of EXO1 increases replication stress, DNA damage, and genomic instability in FANCG- and ZRSR2-deficient cells and support the conclusion that these effects contribute to the selective loss of viability observed in these genetic backgrounds.

### PARP inhibitors olaparib and veliparib have an additive effect on viability of *FANCG* and *ZRSR2* KO cells treated with ART5537 in combination

PARP inhibitors are a leading example of targeted therapies for cancers with DDR deficiencies with established clinical efficacy in HR-deficient cancers [4, 34]. Notably, PARP inhibitors have also been recently explored in clinical trials as a therapeutic option for cancers with mutations in HR genes beyond BRCA1/2, but this approach has largely not been successful. HR deficiencies investigated include FA core complex FANCA deficiency in the combinatorial AMPLITUDE trial [35], where addition of PARP inhibitor niraparib to abiraterone as standard-of-care treatment did not significantly improve patient survival.

We have shown that *EXO1*-*FANCG* and *EXO1*-*ZRSR2* double KO cells are additively sensitive to PARP inhibitors [12], including with the therapeutically approved PARP ‘trapping’ compounds olaparib and talazoparib, and a PARP catalytic inhibitor veliparib. Therefore, we investigated whether an EXO1 inhibitor could be effectively combined with a PARP inhibitor in cancers deficient for FA factors or ZRSR2. To this end, KOs for *FANCG* or *ZRSR2* were induced in eHAP iCas9 cells and cells were treated with either ART5537 or the control compound 30, in combination with either fixed concentration of PARP inhibitor olaparib or veliparib (Supplementary Fig. S4A). As was the case with genetic loss of EXO1, the ART5537 and olaparib combination exhibited a strong effect on cell viability in inducible *FANCG* or *ZRSR2* KO cells (Fig. 5A and B), while the combination of the control compound 30 and olaparib had only a modest effect, which was comparable to loss of FANCG and ZRSR2 and olaparib alone (Fig. 5A and B, and Supplementary Fig. S4B and C). Similarly, we found that inducible eHAP iCas9 *BRCC3* KO and *APITD1/MHF1* KO cells, which exhibited sensitivity to PARP inhibition (Supplementary Fig. S4D and E), can also be further sensitized with ART5537 (Fig. 5C and D). Notably, the combination treatment of ART5537 and veliparib in inducible *FANCG, ZRSR2, BRCC3*, and *APITD1/MHF1* KO cells had a similarly strong effect on cell viability when compared with olaparib (Fig. 5E–H), suggesting that the observed additive effect is due to PARP inhibition and not PARP ‘trapping’ as veliparib is considered to be a weak PARP ‘trapping’ compound [36]. Finally, we confirmed these findings in a constitutive KO system, where both *FANCG* KO and *ZRSR2* KO cells were strongly sensitized with a combination of ART5537 and olaparib (Fig. 5I), highlighting a possible combination therapy approach with an EXO1-specific inhibitor for cancers with FA factor or ZRSR2 deficiencies.

**Figure 5.**
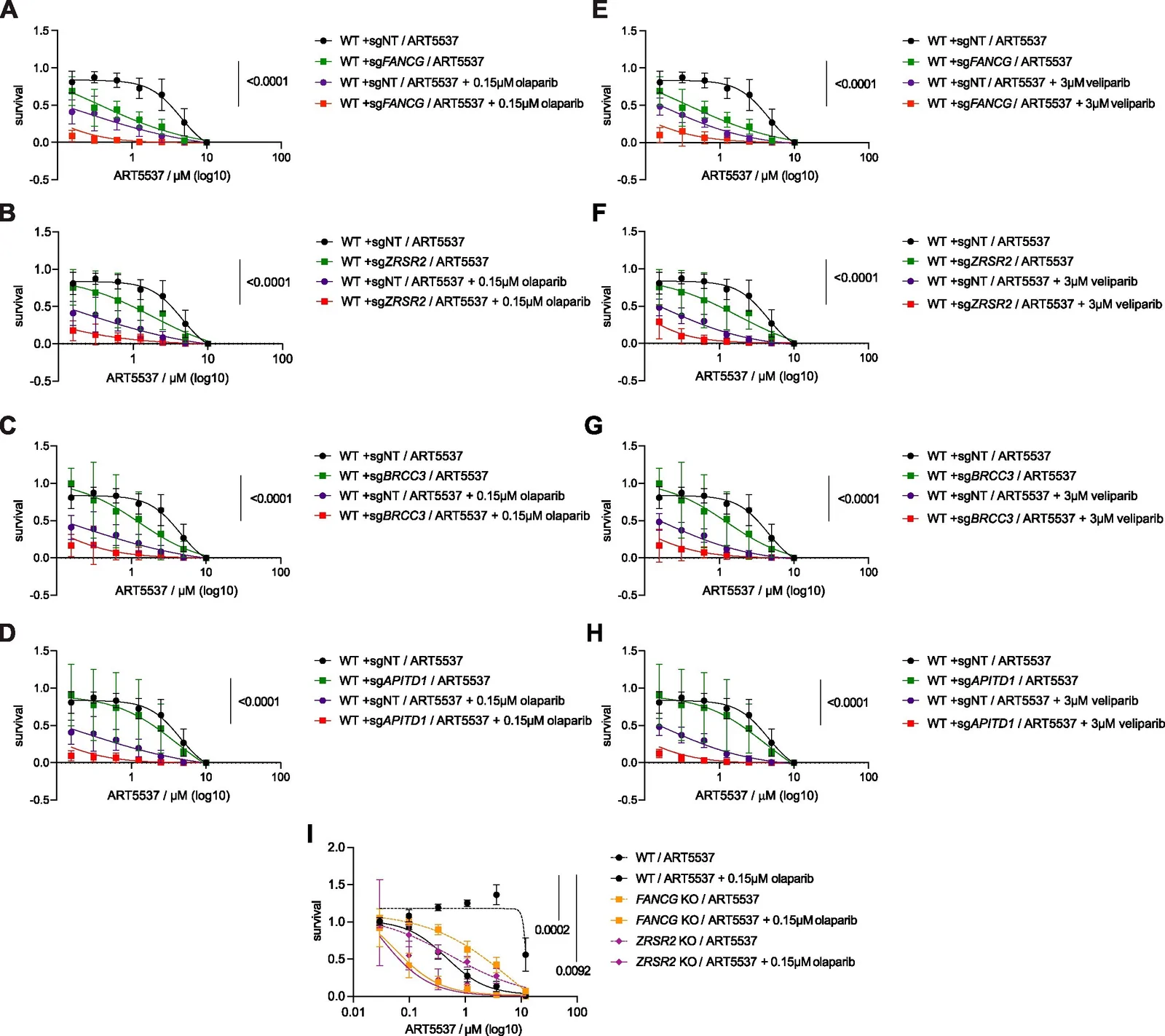
**(A**) Survival of eHAP iCas9 wild-type cells with either sgNT or sg*FANCG* following KO induction after 5-day treatment with either ART5537 alone or ART5537 with 0.15 μM PARP inhibitor olaparib; ordinary two-way ANOVA with Tukey’s test. (**B**) Survival of eHAP iCas9 wild-type cells with either sgNT or sg*ZRSR2* following KO induction after 5-day treatment with either ART5537 alone or ART5537 with 0.15 μM PARP inhibitor olaparib; ordinary two-way ANOVA with Tukey’s test. (**C**) Survival of eHAP iCas9 wild-type cells with either sgNT or sg*BRCC3* following KO induction after 5-day treatment with either ART5537 alone or ART5537 with 0.15 μM PARP inhibitor olaparib; ordinary two-way ANOVA with Tukey’s test. (**D**) Survival of eHAP iCas9 wild type cells with either sgNT or sg*APITD1* following KO induction after 5-day treatment with either ART5537 alone or ART5537 with 0.15 μM PARP inhibitor olaparib; ordinary two-way ANOVA with Tukey’s test. (**E**) Survival of eHAP iCas9 wild type cells with either sgNT or sg*FANCG* following KO induction after 5-day treatment with either ART5537 alone or ART5537 with 3 μM PARP inhibitor veliparib; ordinary two-way ANOVA with Tukey’s test. (**F**) Survival of eHAP iCas9 wild type cells with either sgNT or sg*ZRSR2* following KO induction after 5-day treatment with either ART5537 alone or ART5537 with 3 μM PARP inhibitor veliparib; ordinary two-way ANOVA with Tukey’s test. (**G**) Survival of eHAP iCas9 wild type cells with either sgNT or sg*BRCC3* following KO induction after 5-day treatment with either ART5537 alone or ART5537 with 3 μM PARP inhibitor veliparib; ordinary two-way ANOVA with Tukey’s test. (**H**) Survival of eHAP iCas9 wild type cells with either sgNT or sg*APITD1* following KO induction after 5-day treatment with either ART5537 alone or ART5537 with 3 μM PARP inhibitor veliparib; ordinary two-way ANOVA with Tukey’s test. (**I**) Survival of eHAP iCas9 wild type, *FANCG* KO and *ZRSR2* KO cells after 5-day treatment with either ART5537 alone or ART5537 with 0.15 μM PARP inhibitor olaparib; ordinary two-way ANOVA with Tukey’s test.

## Discussion

In this study, we extend prior genetic observations to demonstrate that pharmacological inhibition of EXO1 is sufficient to selectively compromise the viability of cancer cells harbouring deficiencies in the Fanconi Anaemia (FA) pathway, BRCA1-A complex, or the spliceosome factor ZRSR2. These findings build directly on our earlier work, which identified EXO1 as a synthetic lethal partner of these pathways through genome-wide CRISPR screening and mechanistic interrogation of replication-associated repair defects [12]. Moreover, we have used a potent and selective EXO1 inhibitor to confirm these findings in the context of patient-derived cancer cell lines with FA pathway deficiencies. These findings may also explain sensitivity of other cancer cell lines to pharmacological inhibition of EXO1 [13]. For example, RAMOS, a B-cell lymphoma cell line from a male patient, has a mutation in X-linked gene ZRSR2 (R460G), and has exhibited strong sensitivity to ART5537 both alone and in combination with PARP inhibition [13]. Together, our study provides a framework in support of EXO1 as a conditional dependency in certain replication-stressed cancer.

A key advance here is the demonstration that pharmacological inhibition of the nuclease activity of EXO1 phenocopies genetic loss of EXO1 across multiple DDR-deficient backgrounds, an essential step towards translating genetic studies into the clinic both in the context of EXO1 inhibitors and more broadly. EXO1 is a multifunctional protein, and catalytic inhibition might not be expected to recapitulate scaffolding or protein–protein interaction functions [14]. The close concordance between pharmacological and genetic perturbation argues that nuclease activity is the dominant functional requirement in these contexts, consistent with complementation data showing that catalytic-dead EXO1 fails to rescue synthetic lethality [12]. Mechanistically, both studies converge on a model in which EXO1 processes replication-associated DNA structures that accumulate in FA-, BRCA1-A-, and ZRSR2-deficient cells. In our previous work, this was linked to defective fork reversal, increased fork speed, and accumulation of post-replicative ssDNA gaps. The current study reinforces this model by showing increased ATM-dependent KAP1, CHK2 and H2AX phosphorylation, ATR-dependent RPA32 phosphorylation, and micronuclei following EXO1 inhibition in *FANCG* and *ZRSR2* KO cells.

Our previous study provided evidence that ZRSR2 loss attenuates FA pathway activation, compromises FANCD2 monoubiquitylation, and therefore phenocopies FA deficiency. The current data are largely consistent with this, showing overlapping sensitivity profiles and replication stress phenotypes in both *ZRSR2* and *FANCG* KO cells following EXO1 inhibition. It is not yet clear whether ZRSR2 loss directly impairs FA pathway gene expression via splicing defects, or whether the phenotype reflects broader transcriptional or replication perturbations. Nevertheless, this distinction is important if ZRSR2 deficiency creates a ‘functional FA-like state’ through indirect mechanisms, then EXO1 dependency may extend to a wider class of replication-perturbed tumours beyond canonical FA mutations. Therapeutically, the observation that EXO1 inhibition enhances the cytotoxicity of PARP inhibitors is consistent with prior genetic data showing additive effects rather than strict epistasis. This suggests partially non-overlapping functions, potentially reflecting distinct roles in processing replication-associated intermediates. Indeed, inhibition of TYMS, which we previously showed slows DNA replication forks, was sufficient to suppress the lethality of *FANCG* KO cells treated with EXO1 inhibitor and implies that modulation of replication dynamics could arise as a resistance mechanism.

Finally, the estimated prevalence of EXO1-sensitive genotypes corresponding to ∼8% of all cancers highlights the potential clinical relevance of this approach. However, this figure is likely sensitive to how ‘deficiency’ is defined. Many tumours harbour partial loss-of-function mutations, epigenetic silencing, or pathway rewiring that may not confer the same dependency. Functional biomarkers, such as replication stress signatures or ssDNA gap accumulation, as well as genomic biomarkers arising from the DDR pathway deficiency, may ultimately be more predictive than the genotypic status of the gene alone. While beyond the scope of this study, our data also suggest that EXO1 inhibition may sensitize ATM-deficient cells, which could expand the impact of this therapeutic approach.

In summary, this study provides important pharmacological validation of EXO1 as a synthetic lethal target in replication repair-deficient cancers. At the same time, key mechanistic and translational questions remain, particularly regarding the specificity of replication stress phenotypes, the breadth of responsive tumour contexts, and the therapeutic window of EXO1 inhibition. Addressing these will be critical for determining whether EXO1 can move from a compelling genetic dependency to a clinically actionable target.

## Supplementary Material

zcag029_Supplemental_File

## Data Availability

All data are available in this article and the Supplementary data file.
