# Supplementary material for: EXO1 inhibition selectively kills replication repair-deficient cancer cells
Source: NAR Cancer. 2026 Oct 21;8(4):zcag029. doi: 10.1093/narcan/zcag029 (PMC13684273; doi:10.1093/narcan/zcag029)
Supplement: Supplementary file 1 [file zcag029_supplemental_file.docx]

Supplementary Data

**EXO1 inhibition selectively kills replication repair–deficient cancer cells**

Marija Maric, Sarah E. McClelland and Simon J. Boulton

**Supplementary Figure 1:** (A) Experimental schematic. (B) Immunoblotting for BLM protein levels in eHAP iCas9 cells 72 hours after transfection with siRNA against BLM; non-targeting (NT) siRNA was used for the control sample; alpha-tubulin as control for equal loading. (C) Survival of eHAP iCas9 cells transfected with either non-targeting siRNA or siRNA against BLM and treated with EXO1 compound 20 (n=2).

**Supplementary Figure 2:** (A) Frequencies of mutations and homozygous deletions (data in table extracted from cBioPortal) for genes encoding for FA core complex, FANCM complex, BRCA1-A complex, ZRSR2, CDK11B, LY6G6D, EFNA5 and CYBA. (B) Frequency of homozygous deletions of *FANCG*, *APITD1/MHF1*, *BRCC3* and *ZRSR2* in cancer types from TCGA cohort (data extracted from cBioPortal). (C) Schematic for experiments shown in Figure 2B-D, Supplementary Figure 2D-H and Supplementary Figure 3. (D) Immunoblotting for FANCG protein levels in eHAP iCas9 ‘+sgNT’ or ‘+sg*FANCG*’ cells following 4-day Cas9 expression induction with doxycycline; vinculin as control for equal loading. (E) Immunoblotting for APITD1/MHF1 protein levels in eHAP iCas9 ‘+sgNT’ or ‘+sg*APITD1*’ cells following 4-day Cas9 expression induction with doxycycline; alpha-tubulin as control for equal loading. (F) Immunoblotting for BRCC36 in eHAP iCas9 ‘+sgNT’ or ‘+sg*BRCC3*’ cells following 4-day Cas9 expression induction with doxycycline; vinculin as control for equal loading. (G) ICE Synthego analysis of CRISPR-edited *ZRSR2* locus following 4-day Cas9 expression induction with doxycycline. (H) Representative images of wells from clonogenic assay following treatment with EXO1 compound 30 upon induction of KO in eHAP iCas9 cells with integrated sgRNAs (quantification in Figure 2D).

**Supplementary Figure 3:** (A) Clonogenic assay data analysis for compound 29 (ART5537)- and compound 30-treated cells following FANCG loss in eHAP iCas9 WT or *EXO1* KO cell lines (same experiment as in Figure 2 and Supplementary Figure 2), with normalisation against ‘WT +sgNT/untreated’ condition (n=3); ordinary two-way ANOVA with Tukey’s test. (B) Clonogenic assay data analysis for compound 29 (ART5537)- and compound 30-treated cells following APITD1/MHF1 loss in eHAP iCas9 WT or *EXO1* KO cell lines (same experiment as in Figure 2 and Supplementary Figure 2), with normalisation against ‘WT +sgNT/untreated’ condition (n=3); ordinary two-way ANOVA with Tukey’s test. (C) Clonogenic assay data analysis for compound 29 (ART5537)- and compound 30-treated cells following BRCC3/BRCC36 loss in eHAP iCas9 WT or *EXO1* KO cell lines (same experiment as in Figure 2 and Supplementary Figure 2), with normalisation against ‘WT +sgNT/untreated’ condition (n=3); ordinary two-way ANOVA with Tukey’s test. (D) Clonogenic assay data analysis for compound 29 (ART5537)- and compound 30-treated cells following ZRSR2 loss in eHAP iCas9 WT or *EXO1* KO cell lines (same experiment as in Figure 2 and Supplementary Figure 2), with normalisation against ‘WT +sgNT/untreated’ condition (n=3); ordinary two-way ANOVA with Tukey’s test.

**Supplementary Figure 4:** (A) Schematic for experiments shown in Figure 5A-H. (B) Survival of eHAP iCas9 wild type cells with either sgNT or sg*FANCG* following KO induction after 5-day treatment with either compound 30 alone or compound 30 with 0.15μM PARP inhibitor olaparib (n=3), ordinary two-way ANOVA with Tukey’s test. *P*-value for comparison between eHAP iCas9 wild type cells with either sgNT or sg*FANCG* treated with compound 30 alone is expressed next to the bar on the left, *P*-value for comparison between eHAP iCas9 wild type cells with sg*FANCG* treated with compound 30 with or without 0.15μM olaparib is expressed next to the bar in the middle, while *P*-value for comparison between eHAP iCas9 wild type cells with either sgNT or sg*FANCG* treated with both compound 30 and 0.15μM olaparib is expressed next to the bar on the right. (C) Survival of eHAP iCas9 wild type cells with either sgNT or sg*ZRSR2* following KO induction after 5-day treatment with either compound 30 alone or compound 30 with 0.15μM PARP inhibitor olaparib; (n=3), ordinary two-way ANOVA with Tukey’s test. *P*-value for comparison between eHAP iCas9 wild type cells with either sgNT or sg*ZRSR2* treated with compound 30 alone is expressed next to the bar on the left, *P*-value for comparison between eHAP iCas9 wild type cells with sg*ZRSR2* treated with compound 30 with or without 0.15μM olaparib is expressed next to the bar in the middle, while *P*-value for comparison between eHAP iCas9 wild type cells with either sgNT or sg*ZRSR2* treated with both compound 30 and 0.15μM olaparib is expressed next to the bar on the right. (D) Survival of eHAP iCas9 wild type cells with either sgNT or sg*BRCC3* following KO induction after 5-day treatment with either compound 30 alone or compound 30 with 0.15μM PARP inhibitor olaparib; (n=3), ordinary two-way ANOVA with Tukey’s test. *P*-value for comparison between eHAP iCas9 wild type cells with either sgNT or sg*BRCC3* treated with compound 30 alone is expressed next to the bar on the left, *P*-value for comparison between eHAP iCas9 wild type cells with sg*BRCC3* treated with compound 30 with or without 0.15μM olaparib is expressed next to the bar in the middle, while *P*-value for comparison between eHAP iCas9 wild type cells with either sgNT or sg*BRCC3* treated with both compound 30 and 0.15μM olaparib is expressed next to the bar on the right. (E) Survival of eHAP iCas9 wild type cells with either sgNT or sg*APITD1* following KO induction after 5-day treatment with either compound 30 alone or compound 30 with 0.15μM PARP inhibitor olaparib; (n=3), ordinary two-way ANOVA with Tukey’s test. *P*-value for comparison between eHAP iCas9 wild type cells with either sgNT or sg*APITD1* treated with compound 30 alone is expressed next to the bar on the left, *P*-value for comparison between eHAP iCas9 wild type cells with sg*APITD1* treated with compound 30 with or without 0.15μM olaparib is expressed next to the bar in the middle, while *P*-value for comparison between eHAP iCas9 wild type cells with either sgNT or sg*APITD1* treated with both compound 30 and 0.15μM olaparib is expressed next to the bar on the right.
